# Widely Targeted Metabolomics Profiling Reveals the Effect of Powdery Mildew on Wine Grape Varieties with Different Levels of Tolerance to the Disease

**DOI:** 10.3390/foods11162461

**Published:** 2022-08-15

**Authors:** Huan Yu, Hongyan Li, Rongfu Wei, Guo Cheng, Yongmei Zhou, Jinbiao Liu, Taili Xie, Rongrong Guo, Sihong Zhou

**Affiliations:** Grape and Wine Research Institute, Guangxi Academy of Agricultural Sciences, Nanning 530007, China

**Keywords:** widely targeted metabolomics analysis, powdery mildew, wine grape, quality difference, phenolics

## Abstract

Powdery mildew is an economic threat for viticulture because it not only affects grape yield, but also causes a series of impacts on the qualities of fruit and wine, especially the flavors and various metabolites. Different grape varieties may have different levels of powdery mildew resistance/tolerance and their components of their metabolome are also various. In this study, two wine grape varieties, Guipu No.6 (GP6) and Marselan (Mar) with different levels of powdery mildew tolerance, were used to compare the quality differences in metabolism level by using the widely targeted metabolomics method. The results show that GP6 has a better powdery mildew leaf tolerance than Mar. A total of 774 metabolites were detected by using a UPLC-QQQ-MS-based metabolomics approach, and 57 differential metabolites were identified as key metabolites that were accumulated after infection with powdery mildew in GP6 and Mar, including phenolic acids, flavonoids, terpenoids, stilbenes, lipids, nucleotides and derivatives, lignans and coumarins, and quinones. This finding indicates that the defense mechanisms of grape fruit are mainly associated with phenylpropane-flavonoid metabolism. Specifically, stilbenes had greater variations after powdery mildew infection in GP6; while in Mar, the variations of flavonoids, especially kaempferol-3-*O*-glucuronide and luteolin-7-*O*-glucuronide, were more remarkable. The above results demonstrate that stilbenes may play a more important role than flavonoids in resisting powdery mildew infection in GP6’s fruits, and the drastic variations of these phenolic compounds in different wine grapes after powdery mildew infection might also lead to quality difference in the flavors. This study can provide new insights into the understanding of the cause of powdery mildew tolerance in different grape varieties and the effects on the quality of wine grapes infected with the disease exerted by metabolism level.

## 1. Introduction

The grape is one of the most important fruits in the world. On the one hand, they can be directly consumed as table grapes. On the other hand, it can be used to make wine, raisins and other products. However, for a long time, viticulture has been threatened by fungal diseases [1], which not only affect its yield, but also has a series of impacts on the qualities of fruit and wine, especially their flavors and various metabolites [2,3]. Powdery mildew-infected Cabernet Sauvignon (*Vitis vinifera*) and Sauvignon Blanc (*V. vinifera*) grape berries showed a higher total acidity and a reduction in yield than healthy ones, and the total anthocyanin content of Cabernet Sauvignon was decreased while the concentration of 3-mercaptohexanol was decreased in Sauvignon Blanc [4]. Additionally, for Pinot Noir (*V. vinifera*) grape berries, the fruits infected with powdery mildew experienced increased evolution of volatile ethyl acetate, acetic acid, and ethanol [5]. Hong et al. examined the metabolomics of Chardonnay (*V. vinifera*) grape berries infected with *Botrytis cinerea*, and found that phenylpropanoids, flavonoids, and sucrose were largely degraded while glycerol, gluconic acid, and succinate were markedly produced in the infected berries [6]. Semi-targeted metabolomics analysis for downy mildew-infected leaves of *V. vinifera* cv. Malbec vines showed that there was a general decrease in flavonoid-related metabolites, whereas some stilbenoid concentrations increased upon downy mildew infection [7]. Bunch rot of grape berries caused by multiple fungi resulted in compounds including geosmin, 2-methylisoborneol, 1-octen-3-ol, 2-octen-1-ol, fenchol, and fenchone being detected in the infected grapes [8]. Fungal diseases also cause variation in the wine composition and flavor. When grape sugar ripeness was standardized, the levels of titratable acidity (TA), total phenolics, hydroxycinnamates and flavonoids in Chardonnay juice and wine corresponded with the severity of powdery mildew, while powdery mildew-affected juice and wine presented more unpleasant aromas than the healthy controls [9]. Red wines made from downy mildew-infected Merlot (*V. vinifera*) or Cabernet Sauvignon were marked by intense odors of green, herbaceous figs and cooked fruit notes, while 3-methyl-2,4-nonanedione with cooked fruit notes was found in old Merlot wines made with 20% infected berries, demonstrating mildew’s effect on the ability of a wine to age [10]. In Ferreira et al.’s opinion, pathogenesis-related (PR) proteins play an important role in protection against various diseases but result in adverse effects on the clarity and stability of wines [11].

*Erysiphe necator* causes grape powdery mildew [12], which is one of the predominate fungal diseases in viticulture. Different grape varieties may have different levels of powdery mildew resistance. Generally speaking, *V. vinifera* cultivars applied in viticulture have good quality characteristics but are commonly poor in powdery mildew resistance, while some grape varieties sourced from the grape groups of North America and East Asia have certain powdery mildew resistance [13,14]. The compositions of metabolites in grapes and their wines varied according to the powdery mildew resistances of different grape varieties. Atak et al. reported that the phenolics (gallic acid, catechin, and epicatechin) in leaves of different hybrids/cultivars were changed differently after being infected with powdery mildew and downy mildew. [15]. González-Centeno et al. assessed the wine quality characteristics of nine red bouquet hybrid grape varieties (≥97.5% of *V. vinifera* genome) with resistance to downy and powdery mildews with regard to their phenolic and volatile compounds. These were similar to those of common red monovarietal wines and indicated the nine hybrid varieties presented enough potential to become quality wines [16]. Frioni et al. compared five new pathogen-resistant white grape varieties with regard to their content of sugars and organic acids, and the vatiety named Sauvignon Rytos was thought to suit warm sites because of its earlier veraison, faster sugar accumulation, and higher tartrate [17].

Owing to the promotion of rain shelter cultivation technology and two-crop-a-year cultivation in recent years, southern China has become a booming grape production region, and the quality of winter grapes is much better than that of summer grapes in terms of anthocyanins and soluble solids content [18,19]. However, rain shelter cultivation [20] and the climate in the second half of the year [19] provide a favorable environment for the survival of *E. necator*, including a moderate temperature (20–28 °C) with higher relative humidity (80~90%) [21], weak light intensity (ultraviolet ray), and less rainfall [22], meaning that powdery mildew becomes a serious threat to winter fruit production in this area [23]. Utilizing the natural resistance of plants for breeding is an economic and environmentally friendly means to control plant disease [24]. The breeding strategy involving marker-assisted selection with *Run/Ren* Loci is helpful to obtain new genotypes against powdery mildew [25]. Therefore, it is necessary and valuable to understand the effects of powdery mildew on the quality of grape varieties with different levels of tolerance and promote breeding high tolerance varieties against the disease under this cultivation mode in local areas.

In this study, we compared the quality differences in two wine grapes with different levels of powdery mildew tolerance. Guipu No.6 (GP6, *Vitis* sp.) is a variety collected in the field by Guangxi Academy of Agricultural Sciences and Guangxi Academy of Specialty Crops in China. Previous reports showed that Marselan (Mar, *V. vinifera*) has a poorer powdery mildew tolerance than GP6, and GP6 was not close in its genetic relationship with *V. vinifera* [26,27]. The widely targeted metabolomics method with high throughput (774 metabolites were detected in total) was applied to analyze the differences in the metabolome of the Mar and GP6 fruits infected by *E. necator*, and the results provide new insights into the understanding of the effects on the quality of grape varieties with different tolerance levels against powdery mildew exerted by metabolism level.

## 2. Materials and Methods

### 2.1. Materials

The experiment was performed in the vineyards of the Grape and Wine Research Institute, Guangxi Academy of Agricultural Sciences, located in Nanning, Guangxi Province (22°36′39″ N, 108°13′51″ E). Two grape varieties, namely Guipu No.6 (GP6) and Marselan (Mar), were tested. The vines were managed on rain shelter cultivation and were planted in east–west-oriented rows spaced at 1.6 m (between vines) × 3.0 m (between rows). For this study, GP6 and Mar were grown by applying the vertical trellis system under rain shelter cultivation and conventional field management with micro-sprinkling irrigation. The irrigation was performed with an average of 21.6–36.0 L/vine/day during the whole winter fruit development period on sunny days, while it was performed every other day on cloudy days. The powdery mildew-infected GP6 and Mar grapes were grown using the same cultivation and field management techniques as the controls but without fungicide application during the winter fruit development period. For the two varieties, 36 plants in total were applied with these treatments, fungicide management (FM) or no fungicides (NF). These plants were divided into four groups for this study: GP6-FM, GP6-NF, Mar-FM, and Mar-NF. Each group had three biological replicates and every replicate comprised three plants. During the winter fruit development period(from late August to early January of the next year), the average temperature (T) was 21.05 °C, and the average relative humidity (RH) was 81.40%, meaning that these conditions were favorable for powdery mildew development, especially in October to November (T: 21.26 °C, RH: 82.81%).

### 2.2. Disease Investigation Methods

Using the natural field identification method to carry out investigations into the disease indexes of two wine grape varieties following previous studies [18,28], powdery mildew infection in leaves and berries were observed semimonthly during the winter fruit development period from September 2020 to the harvest period in early January 2021. For the disease index of leaves, the shoots were randomly selected to be investigated, and powdery mildew infection in leaves on the shoots from basal to distal position were recorded. No less than 100 leaves were investigated for every biological replicate. The severity of powdery mildew infection on the leaves was divided into different disease levels according to the percentage of disease spot area (PDSA) in the leaf (Level 0: PDSA = 0; Level 1: 0 < PDSA ≤ 25%; Level 2: 25% < PDSA ≤ 50%; Level 3: 50% < PDSA ≤ 75%; Level 4: 75% < PDSA ≤ 100%). Subsequently, the above investigation results for the severity of disease infection on leaves were converted into the disease index for each treatment:

Disease index of leaves (%) = [∑ (level of disease severity × record number of infected leaves at this level)/(total number of leaves investigated × highest level of disease severity)] × 100.

For the disease incidence of berries, the number of powdery mildew-infected berries among the total number of grape berries was recorded. No less than 300 berries from randomly selected grape clusters were investigated for every biological replicate:

Disease incidence of berries (%) = [(the number of infected berries for each biological replicate)/(total number of investigated berries for each biological replicate)] × 100.

### 2.3. Sample Preparation and Physical and Chemical Index Detection Methods

All samples were collected in the maturity period on 8 January 2021. For each treatment, 270 grape berries were sampled (90 grape berries for each replicate), and attention was given to the upper, middle, and lower parts of each grape cluster, ensuring that collected samples from the FM treatment were healthy berries of GP6 and Mar (GP6-He and Mar-He), while those from the NF treatment were berries of GP6 and Mar infected with powdery mildew (GP6-In and Mar-In). Among the collected samples, 30 grape berries from each replicate were used to determine physical and chemical indexes: fresh weight per berry, number of seeds per berry, seed weight per berry, skin weight per berry, fruit pH, titratable acid (TA) content, and soluble solid content (SSC). A PAL-1 handheld refractometer (Atago, Tokyo, Japan) was used to determine the SSC, a pH meter (INESA, Shanghai, China) was used to measure pH value, and the acid-base titration method was used to determine the TA content.

### 2.4. Widely Targeted Metabolomics Experimental Methods

#### 2.4.1. Sample Preparation and Extraction

The extraction process for metabolite analysis was performed based on a previous report [29]. Twelve samples were freeze-dried by vacuum freeze-dryer (Scientz-100F, Ningbo, China). The freeze-dried sample was crushed using a mixer mill (MM 400, Retsch, Haan, Germany) with zirconia beads for 1.5 min at 30 Hz. Then, 100 mg of the lyophilized powder was dissolved in 1.2 mL of 70% methanol solution and vortexed for 30 s every 30 min, six times. After that, the extracting solution was placed in a refrigerator at 4 °C overnight. Following centrifugation at 12,000 rpm for 10 min, the extracts were filtrated (SCAA-104, 0.22 μm pore size; ANPEL, Shanghai, China) before UPLC-MS/MS analysis.

#### 2.4.2. UPLC Conditions

For the sample extracts obtained in the previous step, an UPLC-ESI-MS/MS system (UPLC, SHIMADZU Nexera X2, Kyoto, Japan; MS, Applied Biosystems 4500 Q TRAP, Foster City, CA, USA) was used to carry out analysis. The specific analysis conditions are described below. UPLC: column (Agilent SB-C18, 1.8 µm, 2.1 mm × 100 mm). The mobile phases A and B were pure water with 0.1% formic acid and acetonitrile with 0.1% formic acid, respectively. The gradient program of A and B was as follows: 0 min, 95:5 (*v*:*v*); 9 min, 5:95 (*v*:*v*); 10 min, 5:95 (*v*:*v*); 11.1 min, 95:5 (*v*:*v*); and 13.0 min, 95:5 (*v*:*v*). The mobile phase flow rate was 0.35 mL per min; the column oven was set to 40 °C; and the injection volume was 4 μL. The effluent was alternatively connected to an ESI-triple quadrupole-linear ion trap (QTRAP)-MS.

#### 2.4.3. ESI-Q TRAP-MS/MS

Linear ion trap (LIT) and triple quadrupole (QQQ) scans were acquired on a triple quadrupole-linear ion trap mass spectrometer (Q TRAP), AB4500 Q TRAP UPLC/MS/MS System, equipped with an ESI Turbo Ion-Spray interface, operating in positive and negative ion mode and controlled by the Analyst 1.6.3 software (AB Sciex, Framingham, MA, USA). The ESI source operation parameters were selected according to previous reports [27]. A specific set of multiple reaction monitoring (MRM) transitions were monitored for each period according to the metabolites eluted within this period.

### 2.5. Analytical Methods

#### 2.5.1. PCA (Principal Component Analysis) and HCA (Hierarchical Cluster Analysis)

Unsupervised PCA was performed using the statistics function prompt within R (www.r-project.org, accessed on 26 February 2021). The data were unit variance scaled before unsupervised PCA. The HCA (hierarchical cluster analysis) results of samples and metabolites were presented as heatmaps with dendrograms. Both PCA and HCA were carried out using the R package (www.r-project.org) [30]. For HCA, normalized signal intensities of metabolites (unit variance scaling) were visualized as a color spectrum.

#### 2.5.2. Differential Metabolites Selected

Significantly regulated metabolites between groups were determined by VIP ≥ 1 and absolute Log_2_FC (fold change) ≥ 1. VIP values were extracted from the OPLS-DA result, which also contained score plots and permutation plots, and were generated using R package MetaboAnalystR [31]. The data were log transform (log_2_) and mean centered before OPLS-DA. In order to avoid overfitting, a permutation test (200 permutations) was performed.

#### 2.5.3. KEGG Annotation and Enrichment Analysis

The KEGG Compound database (http://www.kegg.jp/kegg/compound/, accessed on 26 February 2021) was used to annotate all of the identified metabolites. Then, the annotated metabolites were mapped to the KEGG Pathway database (http://www.kegg.jp/kegg/pathway.html). Pathways with significantly up- or down-regulated metabolites mapped to were then fed into metabolite set enrichment analysis (MSEA), their significance was determined by hypergeometric test’s *p*-values.

## 3. Results and Analysis

### 3.1. Statistics of Disease Occurrence by Infection of Powdery Mildew

**Figure 1:**
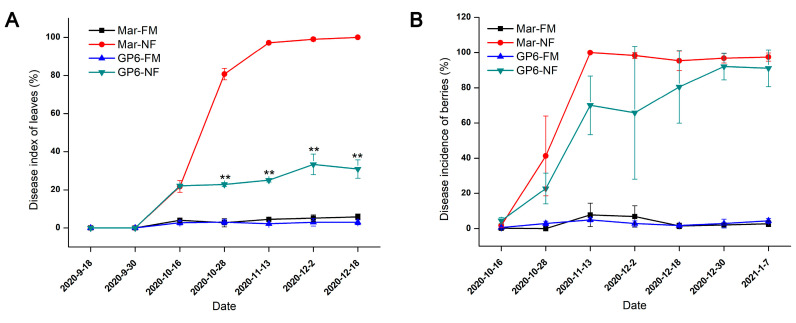
(**A**) Disease index of grape leaves regarding infection with powdery mildew. (**B**) Disease incidence of berries regarding infection with powdery mildew. Data represent mean values, and the bars show standard deviations (*n* = 3). Double asterisks located above a set of symbols indicate a significant difference of *p* < 0.01 compared between Mar-NF and GP6-NF.

The two grape varieties were managed with and without fungicide application, and the occurrence of powdery mildew-infected grape leaves and berries during the whole winter grape development period was investigated semimonthly. The results are shown in Figure 1. The investigation results show that grapes of all four treatment groups were infected with powdery mildew in mid-October after flowering, and NF treatments made this more serious. Before veraison in mid-November, powdery mildew infected rapidly in NF treatments except the leaves of GP6-NF: GP6 exhibited better leaf powdery mildew tolerance than Mar (Figure 1A). Although *E. necator* infected the berries of GP6 more slowly those of Mar, the powdery mildew infection incidence of GP6-NF’s berries was still close to that of Mar during the harvest period in early January, meaning the majority of berries of GP6-NF were also colonized by *E. necator* at that time (Figure 1B). It indicated that the infection of GP6-NF’s berries were just a little slighter compared to Mar-NF.

### 3.2. Physical and Chemical Indexes Analysis

A comparison of the physical and chemical indexes between healthy grape berries and powdery mildew-infected grape berries of the two grape varieties is shown in Table 1. After powdery mildew infection, the fresh weight per berry significantly reduced in two varieties. Skin weight per berry and seed weight per berry were significantly decreased in powdery mildew-infected GP6 grapes. However, there were no significant difference of these indexes between Mar-He and Mar-In. For some other indexes, such as seed number per berry, skin to berry ratio, SSC, pH, and TA, also showed no significant changed between the healthy and powdery mildew-infected grapes. Due to the differences in varieties, the fresh weight per berry, the number of seeds per berry, seed weight per berry, and skin weight per berry were higher in healthy GP6 than those in healthy Mar. For powdery mildew-infected grapes, skin to berry ratio and pH were presented by a higher value in Mar than GP6.

### 3.3. Overview of Grape Berry Metabolites

**Figure 2:**
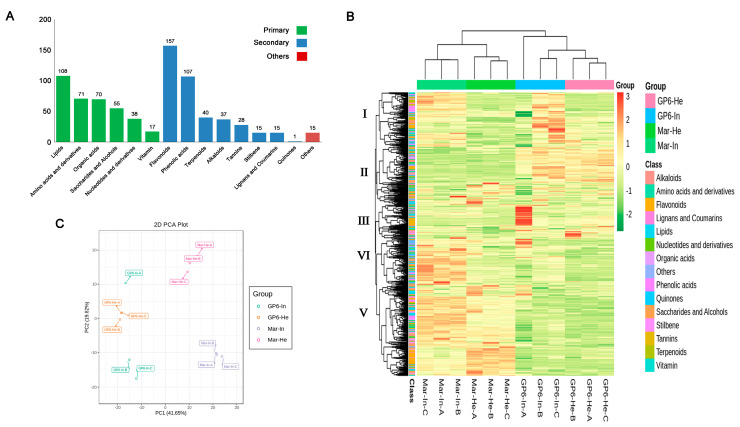
(**A**) Classification of the 774 metabolites of grape berry samples; (**B**) hierarchical cluster analysis (HCA); (**C**) principal component analysis (PCA).

To understand the pattern of metabolite change in Mar-He, Mar-In, in GP6-He, and GP6-In more clearly, the primary and secondary metabolites in the samples were identified using the UPLC-MS platform with widely targeted metabolomics technology. A total of 774 metabolites in 15 categories were detected, including 400 secondary metabolites, 359 primary metabolites, and 15 other substances (Figure 2A, Table S1). The primary metabolites included 108 types of lipids, 71 types of amino acids and derivatives, 70 types of organic acids, 55 types of saccharides and alcohols, 38 types of nucleotides and derivatives, and 17 types of vitamins. The secondary metabolites included 157 types of flavonoids, 107 types of phenolic acids, 40 types of terpenoids, 37 types of alkaloids, 28 types of tannins, 15 types of stilbenes, 15 types of lignans and coumarins, and 1 type of quinones (Figure 2A). The differences in the accumulation patterns of the metabolites in the Mar-He, Mar-In, GP6-He, and GP6-In samples were analyzed by HCA (Figure 2B). The results of this analysis show that there are significant differences in the substances between groups, according to which the metabolites were divided into five clusters. The metabolite contents in Cluster I were the highest in GP6-In, intermediate in Mar-In, and lowest in Mar-He. The metabolite contents in Cluster II were the highest in GP6-In, intermediate in GP6-He, and lowest in Mar-In. The metabolite contents in Cluster III were the highest in GP6-In, intermediate in GP6-He, and lowest in Mar-He. The metabolite contents in Cluster IV were the highest in Mar-In, intermediate in GP6-In, and lowest in GP6-He. The metabolite contents in Cluster V were the highest in Mar-In, moderate in Mar-He, and lowest in GP6-He. In the PCA results, different samples were evidently separated, indicating that the metabolites of different grape varieties experience significant changes after powdery mildew infection, similarly to the differences in physical and chemical indexes. PC1 can explain 41.65% of the characteristics of the original dataset. Different grape varieties were separated by the principal components, while the infected grape berries and healthy grape berries were separated by the second principal components, which explained 19.82% of the characteristics of the original dataset (Figure 2C).

### 3.4. Analysis of Differential Metabolites

**Figure 3:**
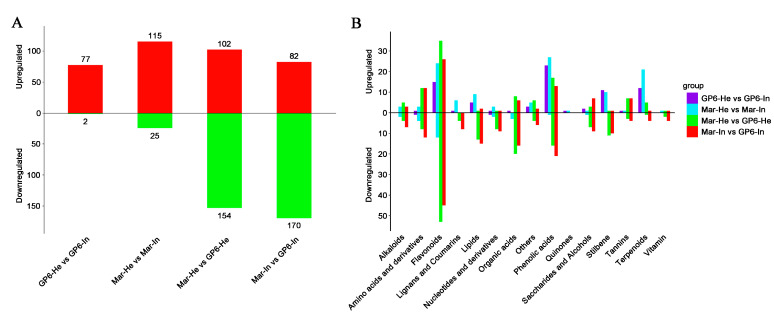
(**A**) The number of differentially expressed metabolites of each pairwise comparison of grapes; (**B**) Classification of differentially expressed metabolites of four pairwise comparisons.

As shown in Figure 3A, in the four comparison pairs (GP6-He vs. GP6-In, Mar-He vs. Mar-In, Mar-He vs. GP6-He, and Mar-In vs. GP6-In) the number of upregulated metabolites was greater than the number of downregulated metabolites. The comparison between GP6-He and GP6-In identified a total of 79 differential metabolites, including 77 upregulated metabolites and 2 downregulated metabolites. The comparison between Mar-He and Mar-In showed a total of 140 differential metabolites, including 115 upregulated metabolites and 25 downregulated metabolites. The comparison between Mar-He and GP6-He found a total of 256 different metabolites, including 102 upregulated metabolites and 154 downregulated metabolites. The comparison between Mar-In and GP6-In showed a total of 252 different metabolites, including 82 upregulated metabolites and 170 downregulated metabolites. As shown in Figure 3B, these differential metabolites were divided into 15 categories, including six primary metabolites (including vitamins, nucleotides and derivatives, saccharides and alcohols, organic acids, amino acids and derivatives, and lipids) and eight secondary metabolites (including alkaloids, flavonoids, lignans and coumarins, phenolic acids, quinones, stilbenes, tannins, and terpenoids). The number of differential metabolites between Mar-He and GP6-He was almost equal to that between Mar-In and GP6-In, and the upregulation and downregulation of the corresponding differential metabolites in the two comparison pairs were similar, determining the characteristics of the grape varieties. The upregulated metabolites between GP6-He and GP6-In and those between Mar-He and Mar-In were all concentrated in flavonoids, phenolic acids, stilbenes, and terpenoids, indicating that these differential metabolites are accumulated more in GP6 and Mar after powdery mildew infection, which might indicate a defense response to the disease.

### 3.5. KEGG Functional Annotation and Enrichment Analysis of Differential Metabolites

**Figure 4:**
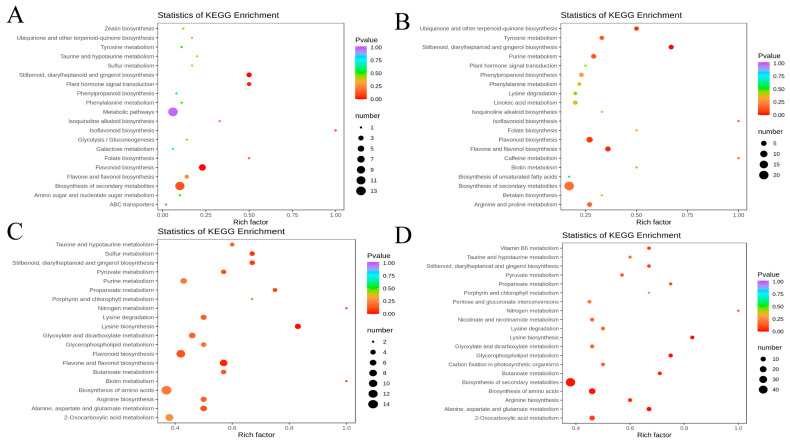
KEGG annotations and enrichment of differentially expressed metabolites of each pairwise comparison of grape berries. (**A**) GP6-He vs. GP6-In; (**B**) Mar-He vs. Mar-In; (**C**) Mar-He vs. GP6-He; (**D**) Mar-In vs. GP6-In.

The differential metabolites between GP6-He and GP6-In were primarily annotated and enriched in the following pathways: biosynthesis of secondary metabolites, flavonoid biosynthesis, stilbenoid, diarylheptanoid and gingerol biosynthesis, plant hormone signal transduction, and phenylpropanoid biosynthesis (Figure 4A).

The differential metabolites between Mar-He and Mar-In were primarily annotated and enriched in the following pathways: biosynthesis of secondary metabolites, flavone and flavonol biosynthesis, flavonoid biosynthesis, stilbenoid, diarylheptanoid and gingerol biosynthesis, ubiquinone and other terpenoid-quinone biosynthesis, purine metabolism, and phenylpropanoid biosynthesis (Figure 4B).

The differential metabolites between Mar-He and GP6-He were primarily annotated and enriched in the following pathways: biosynthesis of amino acids, biosynthesis of secondary metabolites, flavonoid biosynthesis, lysine biosynthesis, stilbenoid, diarylheptanoid and gingerol biosynthesis, sulfur metabolism, and alanine, and aspartate and glutamate metabolism (Figure 4C).

The differential metabolites between Mar-In and GP6-In were primarily annotated and enriched in the following pathways: biosynthesis of secondary metabolites, biosynthesis of amino acids, lysine biosynthesis, glycerophospholipid metabolism, and alanine, and aspartate and glutamate metabolism (Figure 4D).

In these comparison pairs, the overlapped metabolic pathways included flavone and flavonol biosynthesis, flavonoid biosynthesis, stilbenoid, diarylheptanoid and gingerol biosynthesis, ubiquinone and other terpenoid-quinone biosynthesis, and plant hormone signal transduction. Of note, the metabolic pathways mentioned above all pertain to the biosynthesis of secondary metabolites.

### 3.6. Analysis of the Main Differential Metabolic Components

**Figure 5:**
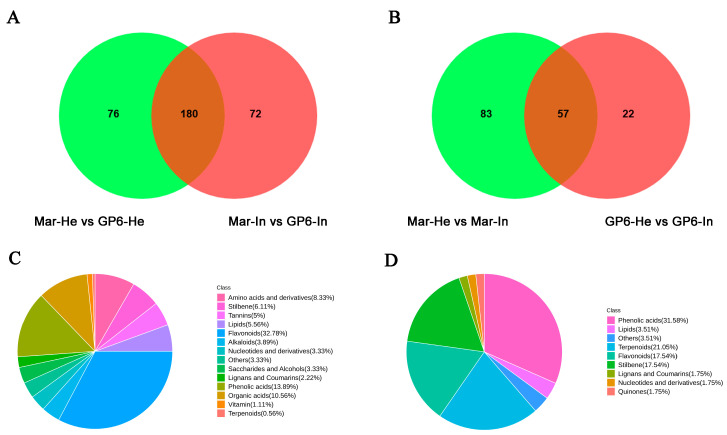
(**A**) Venn diagram between Mar-He vs. GP6-He and Mar-In vs. Gp6-In; (**B**) Venn diagram between GP6-He vs. GP6-In and Mar-He vs. Mar-In; (**C**) The classification of the 180 key metabolites; (**D**) The classification of the 57 key metabolites.

**Figure 6:**
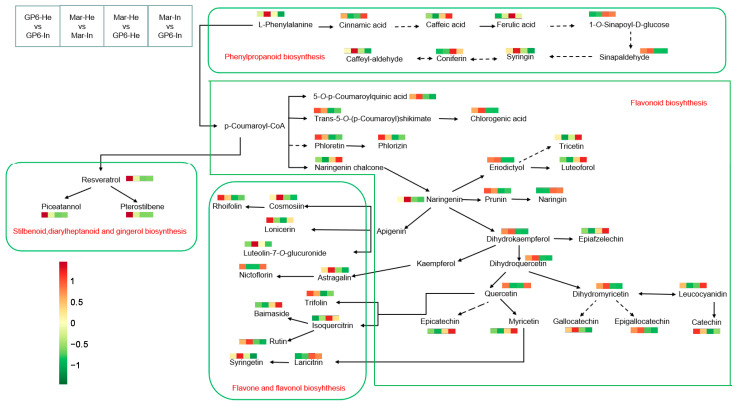
Pairwise comparisons of changes in key metabolites mapped to metabolic pathways in grape fruit. Heatmap colors represent the metabolites expression (log_2_ fold change) for each comparison.

The Venn diagrams depict the differences in differential metabolites between Mar-He vs. GP6-He and Mar-In vs. Gp6-In (Figure 5A), GP6-He vs. GP6-In and Mar-He vs. Mar-In (Figure 5B). Furthermore, the common differential metabolites proportions of Mar-He vs. GP6-He, Mar-In vs. Gp6-In, GP6-He vs. GP6-In, and Mar-He vs. Mar-In are shown in Figure 6C,D. In Mar-He vs. GP6-He and Mar-In vs. Gp6-In (Figure 5A), 180 common differential metabolites were considered to be the key metabolites of variety variation between GP6 and Mar, which mainly included 32.78% flavonoids, 13.89% phenolic acids, 10.56% organic acids, and 5.00% tannins (Figure 5C). In GP6-He vs. GP6-In and Mar-He vs. Mar-In (Figure 5B), 57 common differential metabolites were considered to be the key metabolites produced in GP6 and Mar after powdery mildew infection (Figure 5D), including phenolic acids, terpenoids, stilbenes, flavonoids, organic acids, lipids, nucleotides and derivatives, lignans and coumarins, quinones, and others (Figure 5D).

### 3.7. Common Differential Metabolites of Phenylpropane-Flavonoid Pathways

The common differential metabolites of phenylpropane-flavonoid in two grape varieties are presented by pairwise comparisons in Figure 6. Powdery mildew infection significantly stimulates the production of sinapaldehyde, naringenin, dihydrokaempferol, 5-*O*-*p*-coumaroylquinic acid, trans-5-*O*-(*p*-coumaroyl) shikimate, chlorogenic acid, phloretin, phlorizin, eriodictyol, isoquercitrin, laricitrin, trifolin, and rhoifolin in two grape varieties (Figure 6). Of note, flavan-3-ols, such as gallocatechin, epigallocatechin, and catechin, were also upregulated in infected GP6 and Mar. The major difference between GP6 and Mar is that the significantly upregulated metabolites in GP6 are primarily stilbenes (especially pterostilbene, piceatannol, and resveratrol, which had a fold change exceeding 10), while the significantly upregulated metabolites in Mar are primarily flavonoids (especially kaempferol-3-*O*-glucuronide and luteolin-7-*O*-glucuronide, which had a fold change exceeding 17) (Table S2, Figure 6). Therefore, the defense mechanisms of grape fruit after powdery mildew infection might be associated with the biosynthesis of phenylpropanoid, flavones and flavonols, flavonoids and stilbenoids, and the metabolic pathway map further verified the similarities in the metabolic pathways of grape fruit after powdery mildew infection (Figure 6). On the other hand, no significant differences in anthocyanins were found in the pair comparison between infected and healthy fruits, but the differences in anthocyanins between GP6 and Mar mainly existed in individual metabolites (Table S3). For variety comparison, GP6 has more contents than Mar, such as coniferin, caffeic acid, ferulic acid, 1-*O*-Sinapoyl-D-glucose, naringin, epicatechin, isoquercitrin, baimaside, luteolin-7-*O*-glucuronide, Cyanidin-3,5-*O*-diglucoside, Petunidin-3,5-*O*-diglucoside, and Malvidin-3,5-*O*-diglucoside (Figure 6, Table S3).

## 4. Discussion

Powdery mildew can affect viticulture and fruit quality, which may vary among different grape varieties. However, some responses in grape berries and leaves might be differentially regulated after powdery mildew infection, indicating organ-specific mechanisms [32]. In our study, the powdery mildew tolerance of GP6 leaves was significantly better than that of Mar, while the powdery mildew progressed more slowly in GP6 berries than in Mar berries. However, infected berries of these two grapes had very close disease incidence in the harvest period. The organ-specific tolerance to powdery mildew in leaf and fruit was also overviewed and studied by Zini et al. [33]. Additionally, because of ontogenic resistance to powdery mildew in grape berries [34], if the powdery mildew infection was not severe enough in the early stage, the disease effect to the fruit could not become worse with grape berries’ development.

Powdery mildew has different effects on different physical and chemical indexes of grape fruit. Powdery mildew infection did not affect the fresh weight per berry in some grape varieties [32,35] but reduced the fresh weight per berry in other grape varieties [4,35]. In this study, powdery mildew infection reduced the fresh weight per berry in both varieties. This might be related to fruit shrinkage and desiccation caused by powdery mildew infection [22,36]. On the other hand, powdery mildew infection had no effects on SSC, seed number, skin to berry ratio, SSC, pH, and TA in two grape varieties. For GP6 and Mar, there are differences between the two varieties. The infected berries of GP6 had a lower skin to berry ratio than these of Mar, which might be the basis for the similar degree of infections between them despite higher biochemical tolerance in GP6 with the higher level of upregulation of stilbenes.

Phenolic compounds are one of the most important flavor substances in wine grapes and wines [37]. Phenolics can be divided into flavonoid and nonflavonoid compounds [37,38]. (1) Flavonoid compounds are formed by connecting two aromatic rings through an oxygen heterocycle. According to the degree of hydrogenation and the substitution of heterocycles, flavonoid compounds can be divided into flavonols, flavonoids, isoflavones, anthocyanins, flavanols, etc. Flavonoid compounds usually appear in the form of glycosides in nature. (2) Nonflavonoid compounds are usually called phenolic acids and stilbenes. The synthesis of phenolic compounds acts as the first defensive response against biotic stress and abiotic stress [39]. In this study, the changes in the metabolites of the grape berries from the two varieties after powdery mildew infection were similar: both showed the upregulation of phenolic acids, flavonoids, terpenoids, stilbenes, lipids, nucleotides and derivatives, lignans and coumarins, and quinones. Of note, flavonoids and phenolic acids had the highest proportion of differential metabolites for variety comparison and disease infection. The phenylpropanoid pathway is a rich source of plant metabolites, is indispensable for lignin biosynthesis, and is a starting point for the production of many other important compounds, such as flavonoids, coumarins and lignins [40]. This finding indicates that the defense mechanisms of the two grape varieties after powdery mildew infection may be associated with phenylpropane-flavonoid metabolism.

The phenylpropanoid pathway may also lead to the formation of phenolic acids. In addition to their role in the flavor formation of fruit and wine, free phenolic acids are effective antifungal agents by formation of structural barriers [41]. In this study, phenolic acids were upregulated in the two grape varieties after powdery mildew infection, which confirms that fungal pathogen infection causes changes in phenolic acid metabolism [42,43]. Flavonoid compounds play a role in the entire plant kingdom as UV protective agents, pollinator attractants and antifungal compounds, and they play an important role in the defense of plants against pathogens [43,44]. The prenylation of flavonoid compounds increases their antibacterial and antifungal activities by increasing their lipophilicity and membrane permeability [45]. Lignification may prevent the further spread of *E. necator* [46]. The previous studies have also reported that total phenols, phenolic acids, and flavonoids of Chardonnay grape juice were increased with powdery mildew infection [9]. In summary, the invasion of *E. necator* stimulated phenylpropanoid biosynthesis, flavone and flavonol biosynthesis, flavonoid biosynthesis, stilbenoid, diarylheptanoid, and gingerol biosynthesis in GP6 and Mar, which resulted in the biosynthesis of phenolic acids, flavonoids (flavonols and flavan-3-ols), terpenoids, stilbenes, lignans, and coumarins to resist the spread of powdery mildew. Meanwhile, the increase in these metabolites, especially phenolic acids, flavonoids, and terpenoids, might have an influence on fruit taste, flavor, and potential subsequent wine quality.

Stilbenes are a small yet important class of non-flavonoid polyphenols, various stilbenes have different antifungal abilities, and their main function is to resist powdery mildew [47]. The synthesis pathway of stilbenes pertains to the phenylalanine metabolic pathway [48]. The stilbene content in grape berries was significantly increased after powdery mildew infection [49,50]. The stilbene content after powdery mildew infection can be used to distinguish resistant varieties from susceptible varieties [50]. In this study, the degree of stilbene upregulation after powdery mildew infection was significantly greater in GP6 than in Mar. Meanwhile, it is speculated that stilbenes are one of the more important phenolic compounds that play a role in protecting against pathogens in GP6. On the other hand, in contrast to the fact that the upregulated metabolites in GP6 were primarily stilbenes, the significantly upregulated metabolites in Mar with powdery mildew infection were primarily flavonoids, especially kaempferol-3-*O*-glucuronide and luteolin-7-*O*-glucuronide. The two varieties responded differently to powdery mildew infection, indicating that different varieties developed different defense strategies after powdery mildew infection [51].

Interestingly, various stilbenes have different antifungal abilities, and their main function is to resist powdery mildew [47]. Resveratrol is a phytoalexin with disease resistance in plants, and resveratrol in fruits provides health benefits for humans [52]. In plants, resveratrol can produce derivatives such as polydatin, viniferin, and pterostilbene. The previous study shows that viniferin can respond to grapevine downy mildew [47]. Many studies show that resveratrol, a phytoalexin, can respond to the induction of fungal diseases [47,53,54]. It is worth noting that pterostilbene was the most upregulated in GP6 after powdery mildew infection. Pterostilbene is a methylated resveratrol, which had the same toxicity to *B. cinerea* and *E. necator* [50]. The expression level of stilbenes in the infected site can be used to distinguish sensitive varieties from resistant varieties [50]. Therefore, pterostilbene is possibly a biomarker of powdery mildew infection in GP6.

## 5. Conclusions

Powdery mildew infection can cause changes of physical and chemical indexes, and metabolites of grape fruit. It should be noted that the specific changes in these indicators vary with varieties, which is also the root cause of differences in powdery mildew tolerance among different varieties, and leads to quality differences when wine grape varieties with a different tolerance experience powdery mildew. The tolerance of GP6 to powdery mildew was better than that of Mar. In addition, there was an organ-specific tolerance to powdery mildew of the same variety. In this study, phenolic acids, flavonoids, terpenoids, stilbenes, lipids, nucleotides and derivatives, lignans and coumarins, and quinones were accumulated more in grape fruit after infection by powdery mildew for the two varieties; and among them, flavonoids and phenolic acids had the highest proportion. This finding indicates that the defense mechanisms may be mainly associated with phenylpropane-flavonoid metabolism. Phenolic compounds play a very important role in the flavor of wine grapes and wines. In this study, specifically, stilbenes (especially pterostilbene, piceatannol, and resveratrol) had greater variations after powdery mildew infection for GP6, while in Mar, the variations of flavonoids (especially kaempferol-3-*O*-glucuronide and luteolin-7-*O*-glucuronide) were more remarkable. On the one hand, this result reveals that the above-mentioned metabolites could play roles in the metabolic mechanisms affecting the interaction between powdery mildew and grape fruit, and stilbenes could be more important in resisting powdery mildew than flavonoids in GP6 fruits. On the other hand, the drastic variations of these phenolic compounds in different wine grapes after powdery mildew infection could also lead to quality differences in the flavors. This study can provide new insights into the understanding of the causes of tolerance in different grape varieties and the effects on the quality of wine grapes infected with powdery mildew. It is also helpful to formulate precise strategies and new solutions for the prevention and control of grape powdery mildew based on differences in tolerance.

## Figures and Tables

**Table 1:** Comparison of physical and chemical indexes between healthy berry and infected berry of two grape varieties.

Physical and Chemical Indexes	GP6-He	GP6-In	Mar-He	Mar-In
Berry weight (g)	2.29 *±* 0.08 ^a^	1.52 ± 0.20 ^b^	0.89 ± 0.03 ^c^	0.61 ± 0.04 ^d^
Seed number per berry	2.19 ± 0.08 ^a^	1.88 ± 0.30 ^ab^	1.70 ± 0.17 ^b^	1.61 ± 0.13 ^b^
Seed weight per berry (g)	0.09 ± 0.00 ^a^	0.07 ± 0.02 ^b^	0.05 ± 0.01 ^bc^	0.04 ± 0.00 ^c^
Skin weight per berry (g)	0.36 ± 0.06 ^a^	0.22 ± 0.07 ^b^	0.18 ± 0.03 ^b^	0.14 ± 0.01 ^b^
Skin to berry ratio (%)	15.59 ± 2.34 ^b^	14.42 ± 5.27 ^b^	20.26 ± 3.23 ^ab^	22.72 ± 0.74 ^a^
Soluble solids concentration (°Brix)	21.20 ± 0.10 ^a^	20.10 ± 0.61 ^a^	19.63 ± 0.75 ^a^	19.73 ± 1.76 ^a^
Berry pH	2.72 ± 0.02 ^b^	2.78 ± 0.10 ^b^	2.92 ± 0.03 ^a^	2.91 ± 0.02 ^a^
Titratable acidity (g·L^−1^)	19.23 ± 0.64 ^b^	21.82 ± 3.57 ^ab^	19.89 ± 0.49 ^ab^	23.13±0.84 ^a^

Note: Data represent mean ± standard deviation (*n* = 3). In each row, different letters indicate significant differences among samples according to the Duncan test (*p* < 0.05).

## Data Availability

Data are contained within the article or the Supplementary Materials.

## Supplementary Materials

The following supporting information can be downloaded at: https://www.mdpi.com/article/10.3390/foods11162461/s1, Table S1: the information of all detected metabolites; Table S2: Fifty-seven key differential metabolites; Table S3: Comparison of anthocyanins in the four treatment groups.
